# Book Review

**Published:** 1939

**Authors:** 


### Reviews of Books

**The Surgery of Pain.** By R. Leriche, M.D., LL.D. F.R.C.S. Pp. xx., 512. London: Baillière, Tindall & Cox 1939. Price 21s.—Anything contributed by this eminent Strasbourg surgeon is always fresh, stimulating and based on a wide and very specialized experience. He writes that we have been far too obsessed by the idea of the sensory nerves in thinking about pain. The conception of the physiologists is too mechanical, too purely artificial. There are other factors. Men of some nations, for instance certain Russians and many Asiatics, can tolerate amputation with scarcely any evidence of pain; to those of other races a needle-prick may be intolerable. Vasomotor changes, especially vasoconstriction, greatly increase pain. Division of the sensory root of the Gasserian ganglion is eminently successful in the treatment of neuralgia, but most peripheral neurotomies are failures; the pain sooner or later recurs. Leriche has given up cordotomy, posterior radiotomy and peripheral neurotomies for this reason. The kind of pain he is interested in appears to be independent of the anatomical distribution of the nerves or of nerve roots. Even amputations do not stop it. He believes that such pains travel by the sympathetic system and by the nerves on the arteries. Frequently pain is evoked by the sympathetic without being conducted by it, by producing in the sensory nerve-endings a state of vasoconstriction or of vasodilatation. Pain may also be due to calcium deprivation; the acute agony seen in certain cases of tetany is at once abolished by an injection of calcium. For post-traumatic spreading neuralgia, following, it may be, a trivial injury but leading to pain all over the limb, nerve sections, amputations or periarterial sympathectomy are useless. The proper treatment is, in early cases, novocain injections into the painful area and along the sensory nerves supplying it. Daily injections may be called for. In more extensive cases, novocainization of the stellate and two upper thoracic ganglia on one or two occasions may relieve. Stellectomy does not give much relief. For painful amputation or wound neurogliomata on nerve-trunks, a long excision of the injured nerve should be made and a nerve-graft applied to the cut end. Causalgia (the " burning pain ") is associated with atrophy of the skin, which the patient often insists on keeping wet ; it may follow a partial section of a nerve, or there may be no nerve injury. It is a form of excessive vasomotor reaction to an injury. In early cases, novocainization of the scar and of the stellate ganglion may suffice to cure ; if not, a high periarterial sympathectomy, *e.g.* on the axillary artery, will probably succeed. If an artery has been wounded it should be excised ; if a nerve, the damaged portion should be excised and the gap sutured or nerve-grafted, in addition to the sympathectomy. Painful neuromata in amputation stumps must not be treated by neurectomy, alcohol injections, or re-amputation, but by novocainization of the paravertebral sympathetic or perhaps a ganglionectomy. The " terrible disease " sclerodermia can be cured by parathyroidectomy plus an operation on the sympathetic. Juvenile arteritis is to be treated either by adrenalectomy or by lumbar ganglionectomy. " All cases of angina pectoris should be operated on in the absence of contra-indications." The best treatment is removal of the left stellate ganglion. Leriche lost none of his twenty-seven cases, but not all were cured.

**The Medical Press and Circular, 1839-1939.** Editor: R. J. ROWLETTE, M.D., F.R.C.P.I. Pp. x., 127. Illustrated. London: The Medical Press and Circular. 1939.—The recent celebration by the *Medical Press and Circular* of its first appearance as the *Dublin Medical Press* in 1839 is a matter of great interest in English Medical Journalism. Few other medical papers can point to continuous publication over as long as a century. This extremely interesting record affords us a full account of the development and progress of the paper, especially during its first thirty years before amalgamating the *Dublin Medical Circular.* It is illustrated by a number of reproductions of covers and of portraits of Editors.

**British Medical Societies.** Edited by Sir D'ARCY POWER, K.B.E., F.R.C.S. Pp. xvi., 312. Illustrated. London: Messrs. Baillière, Tindall & Cox. 1939. Price 10s. 6d.—A better title might have been " Some Medical Societies," for, as the Editor himself points out, the list of those noticed is by no means exhaustive, and indeed it is difficult to discover whether any principle guided the selection of the thirty-four societies that do appear. For these include the Society of Apothecaries, a number of Hospital Societies such as Guy's, the Listerian and the Middlesex; amalgamations such as the British Medical Association, the Royal Society of Medicine and the Royal Academy of Medicine in Ireland; some special groups, for example the Ophthalmological, Pathological and Anatomical and, in another class, the Research Defence Society and the Association of Surgeons; and finally a considerable number of the type that is most likely to be suggested by the title, the Medical Society of London, the Hunterian Society, the Edinburgh, York, Manchester, Ulster, and so forth. It is not clear why one Society such as the Cardiff is chosen and another such as our own is omitted. Although naturally the accounts given of the various Societies are very brief, they are with one or two exceptions extremely interesting and informative, particularly in the light thrown on medical habits and modes of thought in earlier times.

**Recollections of Student Life and Later Days.** By C. J. Bond, C.M.G., F.R.C.S. Pp. 47. London: H. K. Lewis & Co. Ltd. 1939. Price 1s.—This is a charming account of the closely associated lives of two great men: of Horsley, the bold thinker, fearless operator and tireless fighter, who died all too soon on "active service" in every sense of the word: and of the modest and retiring Bond of Leicester, the naturalist, philosopher and surgeon, who witnessed and shared in so many of Horsley's triumphs. The two made early acquaintance as students at University College and this ripened into closer friendship as the years rolled by. Horsley's various interests in cerebral surgery, anæsthetics, archæology and sociology are briefly described, and a warm tribute paid to him as a friend and companion. Bond's own interests in parasitology, cardiac physiology, heredity, zoology and botany show what a wide scientific outlook he had, and make it possible to realize how close was the intimacy between these two great men.

**Genetics and the Clinician.** By L. Ride, M.A., B.M., B.Ch., M.R.C.S., L.R.C.P. Pp. xiv., 146. Bristol: John Wright & Sons Ltd. 1938. Price 10s. 6d.—This book, published so far afield as Hong Kong, represents a laudable attempt to present the basic facts of genetics and to indicate the fundamental importance of this rapidly growing branch of biology to the medical profession. It is, as the author points out, very difficult in the crowded medical curriculum to find time for an adequate course on genetics. It therefore behoves the medical practitioner to obtain his knowledge after graduation, and he will find this volume most helpful. A clear account is given of Mendel's original work with the additions made by T. H. Morgan and his school owing to their discovery of "linkage" of genes on the chromosomes, the significance of which is made clear in a chapter devoted to the cytological basis of the mechanism of inheritance. The author then proceeds to the applications of genetics to medicine. Here the relative importance of genetic constitution and of environmental factors—intrinsic and extrinsic forces which interact the one on the other—are considered. Further knowledge on these lines will enable us eventually to formulate sound eugenic methods. The inheritance of specific human characters, both morphogical and physiological, leads the author to a statement on inheritance of serological characters, a subject on which he is an acknowledged authority and one in which knowledge is so exact that it may be applied with confidence to questions involving paternity and, in some cases, identity.

**The Postnatal Development of the Human Cerebral Cortex. Vol. 1 : Cortex of the Newborn.** By J. LEROY CONEL. Pp. viii., 114, iv. Illustrated. London : Oxford University Press (Humphrey Milford). 1939. Price 35s.—This excellent work emanates from the Department of Anatomy of the Boston University School of Medicine and the Department of Pathology of the Harvard Medical School in the Children's Hospital and the Infants' Hospital. The investigation, which is extremely thorough and of high scientific achievement, is intended to be a general survey of the postnatal development of the nerve cells in the normal human cerebral cortex for the purpose of observing what changes occur in the morphology of neurons as development in behaviour of the growing child proceeds. This, the first volume, deals only with the state of development of the neurons in the cerebral cortex of the new-born, *i.e.* the nine-months fœtus immediately after birth. With these objectives, the author has cut and examined many hundreds of microscopic sections of all the principal parts of the frontal, parietal, occipital, and temporal lobes, as well as of the insula and the rhinencephalon, and has passed his findings on to the reader in the form of nearly two hundred very admirable photomicrographs, which are so clear and beautifully reproduced as almost to excel their originals. Le Roy Conel has thus done for the neo-cortex of the new-born brain what von Economo and Koskinas did for the adult neo-cortex in their *Die Cytoarchitekonik der Hirnrinde des erwachsenen Menschen* —a work of surpassing and magnificent opulence—and what, as recently reviewed in this *Journal,* Berry has done for the brain of the mental defective. These three works are thus complementary, and form a mutually dependent trilogy of permanent and undateable scientific value. That Conel's work is not, at the moment, of any great therapeutic interest to either the clinician or the psychologist in no way detracts from its high scientific achievement, for certainly no University Library or Medical School Department, particularly Anatomy, Histology, Physiology, and Neurology, can afford to be without one of the best and most up-to-date expositions of the neuronic construction of the new-born neo-cortex. To them the book can be strongly recommended.

**Treatment in General Practice. Vol. III: Anæsthesia: Surgery.** Articles republished from the *British Medical Journal.* Pp. xii., 402. Illustrated. London: H. K. Lewis & Co. Ltd. 1939. Price 10s. 6d.—This is the third of a series of volumes consisting of articles reprinted from the *British Medical Journal,* and contains forty-two sections, sixteen dealing with anæsthesia and twenty-six with surgery. The scope of the matter contained under each heading is such as may be expected to meet the requirements of the general practitioner, who will find that the various authors have been successful in selecting the most essential points in the topics dealt with. The two chapters by Hewer on the stages and signs and depth of anæsthesia for different operations are illustrative of the practical nature of these articles. Much interesting information is provided under "Analgesia in Midwifery" and other headings, such as "Pre- and Post-Operative Treatment" and "Risks of Anæsthesia." The portion of the volume devoted to surgery is full of useful references to recent methods, including such questions as the use and abuse of antiseptics, the treatment of scalds and burns, the giving of blood transfusion and of saline transfusion; and includes articles describing the methods for dealing with injuries of joints and muscles, lesions of the head, such as scalp wounds or concussion; affections of the eye, ear, nose and throat, and of the teeth. There are illustrations where necessary, and a good index is furnished: the typography is excellent.

**Modern Treatment in General Practice Yearbook, 1939.** Edited by C. P. G. WAKELEY, D.Sc., F.R.C.S., F.R.S.E. Pp. xii., 365. Illustrated. London: Baillière, Tindall & Cox. 1939. Price 12s. 6d.—The *Modern Treatment in General Practice Yearbook* for 1939 maintains the high standard of its predecessor, and in forty-eight articles deals with the modern views on a wide variety of medical and surgical problems. The drawings and photographs are beautifully reproduced and enhance its value; the typography is good. The chief criticism is the inclusion of articles with details of specialized surgical technique, *e.g.* the Technique of Splenectomy, which, in a book of this nature and scope, could have been usefully omitted in favour of contributions more within the sphere of the general practitioner for whom the work is primarily intended. The stimulating section on "Pitfalls in Diagnosis" could with advantage be enlarged. The book is a sound investment for those who are sufficiently interested in clinical medicine and surgery to keep abreast of modern views on these subjects.

**Cancer: Causation, Prevention and Treatment.** By A. E. BLACKBURN, M.D. Pp. viii., 111. Illustrated. London: H. K. Lewis & Co. Ltd. 1939. Price 6s.—The author of this work, an M.D. of Brussels, whose other claim to attention is that he was formerly a Vice-President of the Section of Medicine of the Royal Society of Medicine, has taken his text from the book of Leviticus, where the eating of any animal fat is prohibited; and seeks to persuade us that neglect of this prohibition is responsible for cancer, which he defines as "a vegetable, living parasitically on an animal and striking out roots as vegetables do." He writes of his "theory" and his "researches," though there is nothing worthy of either term in the whole book; which is indeed a mere hotch-potch of unsupported statements and vague speculations interspersed with quotations from any and every source that might be held to support the author's views or to lend a scientific air to the compilation. There are a few obvious misprints, but the value of this contribution to the study of cancer is so completely negligible that the absence of an index may be ignored.

**Iodine and the Incidence of Goitre.** By J. F. MCCLENDON. Pp. x., 126. London: Oxford University Press (Humphrey Milford). 1939. Price 22s. 6d.—This monograph contains an immense amount of information concerning the distribution of iodine in rocks, soil, plants and animals all over the world.

The labour involved in making these thousands of analyses must have been stupendous. The thyroid gland, and seaweeds, contain the richest stores of iodine in nature. Chili saltpetre and certain phosphatic rocks also hold high quantities. The middle section of the book indicates the distribution of goitre all over the world. Certain districts of Switzerland, and the Himalayas, give the highest figure. Only here and there does the author attempt to correlate the incidence of goitre with the amount of iodine available in the food and drink of a district, but in a general way more iodine goes with less goitre, and *vice versa*.

**Rheumatism.** By H. Warren Crowe, D.M., B.Ch., M.R.C.S., L.R.C.P. Pp. xiv., 280. Illustrated. London: John Bale Medical Publications Ltd. 1939. Price 12s. 6d.— This volume is claimed to be for those who, wishing to treat the chronic rheumatic diseases (gout being excluded), will be interested in the theory underlying the most modern technical methods and its practical application as based on the special vaccine treatment of the Charterhouse Rheumatism Clinic. Evidence in the shape of not very convincing photographs, and of a differential sedimentation test, is submitted, but we are not converted. The usual sedimentation test which we relied on for well over ten years is decried, and the method substituted (but not explained) has not the support, to say the least, of all pathologists. The section on Orthopædics, X-ray treatment and Physiotherapy are very good: the book is well produced, printing, paper, illustrations (as illustrations), literary style, are all good. We take off our hats to Dr. Warren Crowe for describing on pages 168–9 the technique for an intrathecal injection and calling it an epidural one.

**The Infant.** Second Edition. By W. J. Pearson, D.S.O., M.C., D.M., F.R.C.P., and A. G. Watkins, M.D. Pp. viii., 56. London: H. K. Lewis & Co. Ltd. 1939. Price 2s. 6d.— When we reviewed the first edition of this book the absence of any advice on the protein-requirement of infants was adversely commented on. In 1939 the importance of adequate protein intake for infants is more generally appreciated than in 1932. On page 12 prominence is given to the caloric requirements of infants, and the caloric yield of protein is given. No word of advice is vouchsafed as to the amount of protein recommended in the artificial feeding of infants. Even more surprising is the avoidance of the topic on page 9, where "modified cow's milk" is discussed and section (*b*) is devoted to "its protein content." The protein requirements of infants are not even mentioned. Analyses of many proprietary foods are given, but this does not atone for the outstanding omission. In the light of present-day knowledge it can scarcely be described now as "a reliable reference book."

**Carbon Monoxide Asphyxia.** By C. K. DRINKER, M.D., D.Sc. Pp. xx., 276. Illustrated. London: Oxford University Press (Humphrey Milford). 1939. Price 25s.—This valuable book is designed to give a practical account of carbon monoxide poisoning for the benefit of those who are concerned with the medical and legal aspects of the condition. The physiology and biochemistry of this form of asphyxia are treated with great clarity. A full account is given of the ætiology, signs, symptoms and diagnosis of acute and chronic monoxide poisoning. The chapter on chronic poisoning is particularly important, since it deals with the question of exposure of garage workers to the exhaust fumes of automobiles. The post-mortem findings in cases of acute poisoning are described adequately, but it is unfortunate that an increase of fluid in the ventricles of the brain is said to be a sign of cerebral œdema. There is an excellent chapter on the treatment of carbon monoxide poisoning, and the final section by Dr. J. Sendroy on the detection and quantitative estimation of carbon monoxide in the air and in the body is a valuable review of the methods available. The book is well illustrated by photographs and diagrams and there is an excellent bibliography which adds very greatly to its value for reference purposes. The index is full and well arranged.

**Fractures and Dislocations.** By J. HOSFORD, M.S., F.R.C.S. Pp. viii., 274. Illustrated. London: H. K. Lewis & Co. Ltd. 1939. Price 12s. 6d.—Within the modest figure of 262 pages, the author has admirably succeeded in covering the essential features and treatments of fractures and dislocations. One feels that perhaps in a subject lending itself so well to illustration by diagrams and photography, practical points in treatment might have been more clearly brought out by a greater use of illustrations. Many important practical points which are not usually recognized are brought out. Some of these are the frequency of refracture in children with a fractured radius and ulna and the wise precaution of prolonging immobilization; the need for almost weekly check X-rays in many cases of fractures of both bones of the forearm to detect insidious slipping; the importance of displacement of the fragments and the need for reduction in many cases of fractures of the carpal scaphoid; and finally, the need for the flexed position in many cases of Colles's fracture. This compact book provides the reader with a well accepted treatment for almost all the fractures and dislocations with which he may be faced.

**Clinical Electro-Surgery.** By G. M. BLECH, G.C.S., G.C.G., M.D., LL.D. Pp. xxviii., 389. Illustrated. London: Oxford University Press (Humphrey Milford). 1939. Price 21s.—One of the Hippocratic aphorisms, probably of Hindu origin, says that "diseases not curable by iron are curable by fire." Much human suffering has resulted from this conception; the present volume is designed to show circumstances where it may, after all, be the truth. The virtues of electro-surgery are that the heat seals lymphatics and capillaries and so prevents the local dissemination of cancer cells; it reduces bleeding; reduces shock and post-operative pain; and promotes wound-healing, though skin sutures should be left in a few days longer than after scalpel incision. It is not claimed that it could be relied on to kill all bacteria in the wound. The main indications are for the purpose of removing malignant growths, for the opening of abscesses, for reducing bleeding when operating on a patient with the hæmorrhagic diathesis, and for sealing off the surface of the thyroid gland to prevent post-operative toxæmia. Bones, nerves and arteries must be avoided. It is better to make the skin incision with a scalpel. In his later chapters the author considers each part of the body *seriatim*, and discusses the very numerous conditions for which electro-surgery may be used. He makes a strong plea, for instance, for this method of operating for carbuncles, for cancer of the breast, and for epithelioma of the vulva. It is maintained that certain toxic goitres, where operative removal would be dangerous, may be exposed under local anæsthesia and treated by a combination of electro-coagulation and electro-excavation, and that this method is both safe and effectual.

**Studies on the Physiology of the Eye.** Reissue. By J. G. BYRNE, M.D., LL.D. Pp. xiii., 440. Illustrated. London: H. K. Lewis & Co. Ltd. 1938. Price 40s.—This book is by a physiologist of international repute in research circles, whose investigations into the special senses have been predominantly in the realms of the eye and ear. With the exception of Part I —about a quarter of the book—which was published in *The American Journal of Physiology*, 1921–29, the experimental studies are here made public for the first time. The experiments on "the functions of the cervical sympathetic as manifested in its action currents" were carried out in collaboration with Professor Willem Einthoven at Leiden University, but apart from this the whole work is the product of the author's own thought and labour. The purpose of the book is to trace the evolution of the multiple and highly complex ocular effector mechanisms in their different stages. The author describes at length the widening and narrowing of the palpebral fissure and the pupil, proptosis and retraction of the eye, and changes in the curvature of the lens. The various reflexes are meticulously divided into component parts, some of which the keen student can easily test for himself, such as preliminary dilatation of the pupil before contraction to light, a phenomenon which has been recorded by flash-lamp photography. An attempt has been made to give clinical significance where possible to the many phenomena elaborated, the keynote of the whole work being the interaction of the parasympathetic and sympathetic impulses and their relation to peripheral nerve stimuli. There is a very interesting section on sleep, hibernation, and the "still reaction" (death-feigning, etc.), and the influence of psychic elements is discussed. So much of the work is pioneer that comparisons and estimations of worth are difficult. But the author's work in London, Paris, Hamburg, Madrid and other university centres has always commanded attention, and his reasoning is impressive. This is an excellent book, but its appeal will be mainly to the physiological research worker and to those clinicians, especially ophthalmic, who delight in profound knowledge for its own sake.

**The Physiology of Anesthesia.** By H. K. BEECHER, A.M., M.D. Pp. xiv., 388. New York: Oxford University Press. 1938. Price 17s. 6d.—Dr. Beecher has produced a valuable digest of many of the experimental data referring to anæsthesia which have been published during the past fifty years by workers in various countries, especially in the United States,

Canada and Europe. The response of the body to anæsthetic agents presents such a wide field for investigation, that the author is to be congratulated on the clear account he has given of the various phenomena involved. To all who are interested in the practice or study of anæsthesia this book will prove invaluable. All aspects of the subject have been considered, with especial attention to the action of the newer drugs and to controversial topics. In the section dealing with the action of local anæsthetics upon various tissues an account is given of recent experiments carried out to determine the factors controlling the level to which spinal anæsthesia will extend: in this connection the occasional occurrence of unaccountable variations makes it obvious that further information is necessary in order to avoid accidents. Throughout the book Dr. Beecher has devoted most attention to such topics as require further clinical consideration. Under the functional effect of anæsthesia upon respiration and circulation there are explanations of many phenomena with which the clinician is only too familiar. But among the organic changes produced by anæsthetics are some effects which may easily be overlooked, as, for instance, the anæmia following repeated Evipan anæsthetics in dogs ; also the damage to the liver in certain cases. The book is beautifully produced, and the references are up to date and will be helpful for research workers and clinicians. A good index is included.

**Gould's Pocket Medical Dictonary.** Eleventh Edition. Edited by C. V. Brownlow. Pp. viii., 1066. London : H. K. Lewis & Co. Ltd. 1939. Price 10s. 6d.—Those readers who are familiar with Gould's *Pocket Medical Dictionary* will welcome this new edition. It contains one outstanding improvement : all words with *prefixes*, *suffixes* and all the *phobias* are now placed in the appendix ; so, too, are the tables of arteries, bones, muscles and nerves, and various other important posological, chemical and bacteriological tables. In some future edition a table of veins will no doubt be included. Arteries are important, of course, in anatomy examinations, but in clinical medicine the veins begin to matter. Another possible improvement would be the collecting of " signs " into the appendix, or even all personal names. Gould's *Pocket Medical Dictionary* has a well-deserved world-wide reputation.

